# Antivirals in COVID-19: A Focus on Pediatric Cardiac Patients

**DOI:** 10.1155/cjid/4573096

**Published:** 2025-03-30

**Authors:** Dalia Safi, Farah Khouri, Rana Zareef, Mariam Arabi

**Affiliations:** ^1^Faculty of Medicine, American University of Beirut Medical Center, Beirut, Lebanon; ^2^Department of Pediatric and Adolescent Medicine, American University of Beirut Medical Center, Beirut, Lebanon; ^3^Division of Pediatric Cardiology, Department of Pediatric and Adolescent Medicine, American University of Beirut Medical Center, Beirut, Lebanon

**Keywords:** antivirals, coronavirus, COVID-19, SARS-CoV-2

## Abstract

The COVID-19 pandemic created an unprecedented public health crisis, driven by its rapid global spread and the urgent need for worldwide collaborative interventions to contain it. This urgency spurred the search for therapeutic agents to prevent or manage the infection. Among these, various types of antivirals emerged as a prominent treatment option, supported by a wealth of observational studies and randomized controlled trials. The results from such studies conflict, with some concluding efficacy and others the lack thereof, with variability also occurring depending on the severity of COVID-19 in the studied population. In addition, many agents have been explored using randomized controlled trials—the gold standard in evaluating the efficacy of an intervention—to only a limited degree, with most of the evidence behind their use concluded using observational studies. Thus, the sheer volume of data has made it challenging to resolve inconsistencies and determine true efficacy. Furthermore, there is a paucity in the literature regarding the use of antivirals in the pediatric population infected with COVID-19, with their use being extrapolated from the results of studies done on adult patients. As such, additional trials are needed to solidify the effectiveness of antivirals in managing COVID-19, particularly in the underexplored and especially vulnerable pediatric cardiac patients. Therefore, utilizing the results from randomized controlled trials, this narrative review evaluates the rationale behind the use of antivirals, summarizes the findings from the literature, and concludes with a focused discussion on their application in pediatric cardiac patients.

## 1. Introduction

The coronavirus disease 2019 (COVID-19) pandemic began in Wuhan, China, driven by a novel coronavirus termed severe acute respiratory syndrome coronavirus 2 (SARS-CoV-2). SARS-CoV-2 is a member of the Coronaviridae family of RNA viruses, from which two other notable viruses have led to widespread morbidity and mortality—SARS-CoV, whose outbreak spanned 2002 and 2003, and MERS, whose outbreak occurred in 2012, with SARS-CoV-2 showing marked homology to the former [1–3]. With 776 million cases and 7.1 million deaths worldwide as of August 2024, the virus has led to global devastation [4]. Severe COVID-19 is characterized by a hyperinflammatory state that may culminate in acute respiratory distress syndrome (ARDS), systemic inflammatory response syndrome (SIRS), shock, and cardiac failure [2]. With the public health burden of the disease and the severity of patient outcomes, the need for effective treatment and prevention became immediately apparent, sparking discourse regarding pharmacological treatments [5, 6].

The current standard of treatment involves symptomatic treatment and supportive care [7]. Furthermore, researchers have been exploring a variety of targeted pharmacological agents and evaluating their efficacy in treating the wide spectrum of presentations. Treatments fall under two main categories—those that target the virus and those that target the host. Drugs that target the virus include antivirals as well as agents with antiviral properties, such as chloroquine and interferons, with varying degrees of success [8, 9]. Treatments targeting the host include immunomodulatory and anti-inflammatory agents, including neutralizing monoclonal and polyclonal antibody therapy such as tocilizumab, convalescent plasma therapy, Janus kinase (JAK) inhibitors like baricitinib, vasoactive intestinal polypeptide analogs like aviptadil, anticoagulants, and corticosteroids [6, 8, 9].

With the advent of vaccines effective against SARS-CoV-2 in preventing symptomatic infection and severe COVID-19, the emergence of novel variants has raised concerns about whether the current vaccines will remain efficacious against the rapidly evolving virus [10–12]. Coronaviruses bearing the largest known genome of RNA viruses have significant plasticity with high rates of RNA recombination, utilizing an RNA-dependent RNA polymerase (RdRp) with low fidelity. Thus, SARS-CoV-2 continues to acquire mutations, raising the need for pharmacological treatment [13, 14].

Antivirals in particular have been explored in a variety of observational studies and randomized controlled trials to test hypotheses based on in vitro studies or rationale stemming from the virus's microbiology with varying results [15]. While some studies conclude the efficacy of certain agents, others find a lack of efficacy instead, leading to conflicting conclusions between observational studies and randomized controlled trials and among different randomized controlled trials. Variability among the results of studies involving patients with varying severity of COVID-19 adds an additional layer of complexity. Furthermore, most studies have been conducted exclusively on the adult population. Hence, the rationale behind the current use of antivirals in the pediatric population stems solely from these studies when antivirals approved for adults may potentially be unsafe or ineffective in children [16]. Pediatric cardiac patients are a particularly vulnerable and underexplored population that would benefit from antivirals that are proven to be effective and safe.

With studies in adults being conflicting and in children being insufficient, this narrative review compiles a broad spectrum of antivirals utilizing the results from randomized controlled trials to settle inconsistencies and critically point out gaps to be filled by clinical trials that would guide future research, especially regarding the underinvestigated pediatric cardiac patients. Hence, this article aims to examine the role of various classes of antiviral drugs in the management of COVID-19. It explores the development and approved clinical applications of these medications, delves into their pharmacological properties and mechanisms of action, and evaluates their efficacy across different stages of COVID-19 infection, from mild to severe cases. Additionally, the article highlights both the therapeutic potential and the challenges associated with their use.

## 2. Methodology

The literature search, conducted until October 28, 2024, involved the use of three databases—Google Scholar, PubMed, and Ovid Medline. The search strategy utilized the following keywords and Medical Subject Headings (MeSH) terms combined as follows: (“COVID-19” OR “SARS-CoV-2”) AND (“Antivirals” OR “Barcitinib” OR “Camostat mesylate” OR “Umifenovir” OR “Ensitrelvir” OR “Lopinavir” OR “Nirmatrelvir” OR “Favipiravir” OR “Molnupiravir” OR “Remdesivir” OR “Ribavirin” OR “Sofosbuvir” OR “Amantadine”).

The articles included cover epidemiology, pathogenesis and pathophysiology, clinical manifestations, risk factors, and treatments for COVID-19. Furthermore, an emphasis was placed on the different aspects of the drug, including its pharmacological parameters, mechanism of action, and use both historically and in COVID-19, with the latter centered specifically on drugs studied in randomized controlled trials published between 2020 and 2024 on COVID-19 patients. Exclusion criteria included drugs that were not extensively studied in a variety of randomized controlled trials as well as agents that are not within the scope of this review, such as corticosteroids, immunomodulatory therapy, antibodies targeting the virus, or drugs that do not primarily have antiviral properties, such as hydroxychloroquine, azithromycin, and ivermectin. In addition, data from ongoing clinical trials were excluded. Only papers written or translated into English were included.

## 3. Antiviral Medications

Antiviral medications are a category of drugs targeted to combating viral infections, as viruses cause a substantial number of human maladies, leading to significant morbidity and mortality throughout the centuries. With the current century marked by an accelerated rate of emergence of microbial infections due to globalization and rapid climate change, there is a continuous need for antivirals [17]. Idoxuridine, the first commercially available antiviral, was synthesized in 1959 as an antitumor agent but was later repurposed for treating herpetic eye infections [18, 19]. This was followed by the drug methisazone, dating back to the 1960s when it was used for treating and preventing smallpox, and later for targeting herpes simplex virus (HSV) and influenza viruses [19].

The history of antiviral development is marked by successes, such as developing a cure for HCV, and failures, with no antivirals effective against poliovirus and hemorrhagic fever viruses [19, 20]. Antivirals comprise a wide variety of biochemical structures targeting different aspects of viral lifecycles [18, 21]. Examples of successful drug targets include the viral DNA or RNA polymerases and proteases. With the goal to halt viral replication in the host comes the significant obstacle to the development of antivirals: resistance, an issue that is especially challenging for RNA viruses due to their error-prone replication, ability to undergo recombination, and quick replication rate [22]. An overview of the traditional indications of the antivirals used in COVID-19 can be found in Table 1.

### 3.1. Pharmacology

With many of the antivirals utilized for COVID-19 being drugs that have other indications, data on the pharmacological parameters stem from studies done based on each drug's respective indication. Many of the drugs are orally bioavailable, and a variety of half-lives support dosing once daily, twice daily, or three times daily. While many of the antivirals are renally cleared, the adjustment of the dose or utilizing a different agent remains an option for those with renal impairment. For the details on the pharmacological parameters of each antiviral agent, refer to Table 2.

### 3.2. Mechanisms of Action

**Figure 1:**
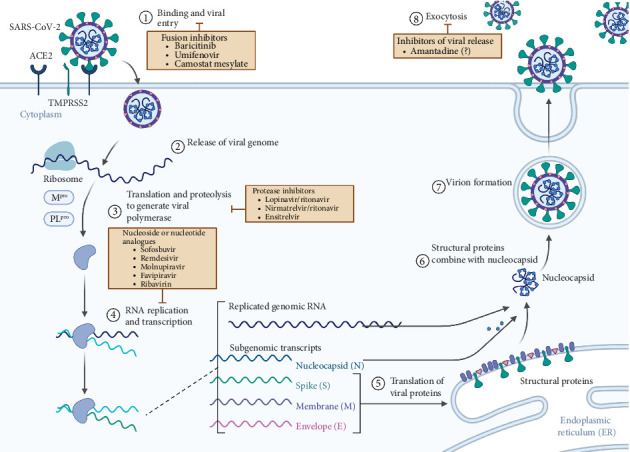
SARS-CoV-2 lifecycle with antivirals targeting specific steps. Goldman-Israelow, Benjamin; Fulford, Ginny (2024). Adapted from “Coronavirus Replication Cycle (Simplified).” Retrieved from https://app.biorender.com/biorender-templates. SARS-CoV-2 is a single-stranded, positive-sense RNA (+ssRNA) virus responsible for the COVID-19 pandemic, which originates as an infection of the respiratory tract [166]. Belonging to the family Coronaviridae, SARS-CoV-2 is classified under the genus *Betacoronaviruses* (BetaCoV), and given that its viral genome is +ssRNA, it can directly act as a messenger RNA (mRNA) to be immediately translated by host cells' translation machinery into viral proteins. Those viral proteins include a total of four structural (phosphorylated nucleocapsid (N), membrane protein (M), envelope protein (E), and surface protein (S)) and 16 nonstructural proteins (Nsps). The S protein is especially important in the virus' ability to infect and is made up of two subunits: S1 and S2 whereby S1 is involved in the initial recognition and binding to its receptor and S2 mediates the fusion of the virus with the membrane of the host cell. Together, the M, E, and S proteins make up the envelope of SARS-CoV-2 and are glycoproteins [167]. Early during infection, the life cycle of SARS-CoV-2 is recognized to follow five stages, the first of which is attachment followed by penetration, biosynthesis, maturation, and terminating in release. The initial stage is mediated by the S protein's specific binding to the host cell receptor—angiotensin-converting enzyme 2 (ACE2)—followed by the second stage of cleaving the S protein into its subunits, S1 and S2, by the host cell's own transmembrane serine protease 2 (TMPRSS2). At this point in time, the S1 subunit functions to continuously switch between two states—open, configured upright before fusion then closed, configured down after fusion—effectively protecting the virus from antibodies by neutralizing them while bound to ACE2. The S2 subunit drives the fusion process, which primarily involves the subunit's fusion protein (FP) disrupting the host cell's membrane, fusing the viral envelope's lipids with those of the host cell membrane. Successfully internalized, the virus now releases its genome into the cytoplasm after fusing with the endosome. This release involves the two major viral proteases: 3CL pro (M-pro, main protease) and the papain-like protease (PLpro), which are involved in cleaving the viral polyprotein to release the 16 Nsps. The third stage commences with the replication of the (+) RNA strand first into a (−) RNA strand, which subsequently undergoes one of two events: replication into a (+) RNA strand for further assembly of virions or for transcribing subgenomic mRNA later translated into the various viral proteins. Translation of the N protein occurs in the cytoplasm, and in the fourth stage, maturation of the M, E, and S proteins localizes at the rough endoplasmic reticulum (RER). This is followed by N protein enclosing the (+) RNA strand previously synthesized, resulting in the nucleocapsid. Full assembly of viral particles with the nucleocapsid takes place in the compartment known as the endoplasmic reticulum-to-Golgi intermediate compartment (ERGIC). Newly synthesized virions are packaged into vesicles in the Golgi that later fuse with the plasma membrane as they are released via exocytosis, terminating the fifth stage [167–169].

The development of antiviral treatment for COVID-19 patients ensued with the purpose of ameliorating the symptoms as well as preventing infection through targeting different levels of its lifecycle [61]. Such antivirals include the following classes: fusion inhibitors, protease inhibitors, nucleoside/nucleotide reverse transcriptase inhibitors (NRTIs), and M2 ion-channel blockers. An overview of SARS-CoV-2's lifecycle along with the selected antivirals' targets can be found in Figure 1. Each drug's mechanism of action is summarized in Table 1.

#### 3.2.1. Fusion Inhibitors

Fusion inhibitors have been shown to have a variety of mechanisms by which they inhibit viral entry into host cells from inhibiting viral receptor-mediated endocytosis to modification of endocytic acidification [23, 32, 33]. The three main antiviral agents that fall under this category are baricitinib, umifenovir (arbidol), and camostat mesylate, discussed further in Table 1.

#### 3.2.2. Protease Inhibitors

In SARS-CoV-2 and many other viruses, protein maturation through viral protein cleavage by protease enzymes is a critical step in their lifecycle, shining light on the possibility of drugs that might inhibit several parts of this stage. The major proteases of SARS-CoV-2, its main protease (M-pro or 3CL protease) and papain-like protease (PL-pro), cleave newly translated proteins into their nonstructural, functional products and thus bear a major responsibility in ensuring successful viral replication [61, 170]. Given that M-pro was initially found to be highly conserved and not detected in humans, drugs targeting this enzyme (M-pro inhibitors) have been grouped into those that covalently inhibit the enzyme and those that perform inhibition noncovalently. Both types target the M-pro catalytic site: The covalent type covalently binds specific amino acids of M-pro with vital activity leading to functional inactivation, whereas the noncovalent type varies in its interactions with the catalytic site. Moreover, most of the inhibitors are of the noncovalent type yet still possess a lower activity profile compared to covalent inhibitors [170]. One direct M-pro inhibitor approved for COVID-19 treatment is the combination of nirmatrelvir/ritonavir whereby ritonavir functions to inhibit the cytochrome P450-3A4 enzyme responsible for metabolizing the active M-pro inhibitor nirmatrelvir. However, recent studies have revealed that M-pro is capable of mutating with specifically identified mutations that allowed resistance development against a class of drugs targeting it, including nirmatrelvir, underscoring the necessity of continuous examination of M-pro inhibitors among circulating coronavirus strains [61].

#### 3.2.3. Nucleoside and Nucleotide Analogs

As small molecules working to inhibit the SARS-CoV-2 RdRp required for viral replication and transcription, nucleoside analogs (NAs) work in the same way nucleotide analogs do and are similar in structure to the natural substrate of RdRp: nucleoside triphosphate (NTP) albeit with specifically unique chemical substitutions [70]. In general, nucleoside/nucleotide analogs are administered as prodrugs for uninterrupted access into cells after which they are converted by host cellular enzymes into their active 5′-triphosphate (TP) form, mimicking the natural NTP [67]. There exist a wide variety of the types of modifications possible in NAs from 1′-ribose modification to base modifications. As such, RdRp may erroneously identify the NA as NTP and actively incorporate it into the primer strand. Upon incorporation, NAs inhibit further SARS-CoV-2 replication and transcription. The major identified mechanisms of RdRp inhibition include immediate and delayed chain terminations as well as mutagenesis and template-dependent termination [70].

#### 3.2.4. M2 Ion-Channel Blockers

The M2 ion-channel protein is usually essential for maintaining pH across the entire viral envelope, and this is critical during the process of cell entry as well as viral movement across the host cell's trans-Golgi membrane during the maturation phase. It is well known that the blockade of this channel is one target used against influenza viruses. In the context of COVID-19, not much research has yielded evidence regarding this mechanism in SARS-CoV-2; however, the initial consensus is that amantadine's structure allows for potential inhibition of channel activity of some protein-membrane channels found in SARS-CoV-2. This process allows for the reduction of the effect of these channels on viral release [17, 132].

## 4. Pros and Cons

**Figure 2:**
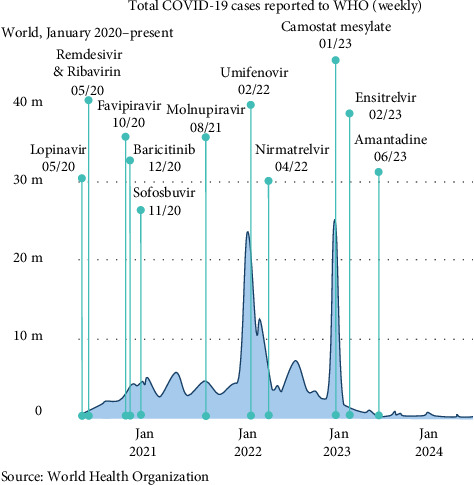
A timeline of the publishing date of the earliest RCTs assessing each antiviral superimposed on incident COVID-19 cases, adapted from the WHO COVID-19 dashboard [4].

Each antiviral has been studied in a variety of contexts in RCTs, with some studies verifying the drug's efficacy while others finding no significant difference. This section will consider RCTs for six of 12 of the most studied antivirals. Tables 3 and 4 provide an overview of selected high-impact studies and their primary outcomes.  Table 5 summarizes findings from RCTs on the remaining six antivirals.  Figure 2 dates the earliest RCTs associated with each of the discussed antivirals.

### 4.1. Remdesivir

Remdesivir is one of the most studied antiviral agents whose trial journey began in 2020 with an RCT concerning adults with severe COVID-19. The study revealed no significant clinical benefit in already hospitalized patients but called for larger trials [68]. A slightly more recent and pivotal RCT known as the Adaptive COVID-19 Treatment Trial-1 (ACTT-1) reported the superiority of remdesivir to placebo in its effect on significantly shortening the time needed for recovery in 1062 COVID-19 patients who were hospitalized and had evidence of lower respiratory tract infection. The results of ACTT-1 were vital in remdesivir achieving Emergency Use Authorization (EUA) by the FDA whereby it was the first antiviral agent recommended for the treatment of COVID-19 based on the improved time for recovery in moderate-to-severe cases [71]. Similarly, a large clinical trial found that remdesivir was associated with an 87% reduction in hospitalization or death in high-risk patients, with an acceptable safety profile [72]. Regarding its adverse effects, remdesivir was not found to be significantly associated with adverse cardiac events contrary to safety concerns about remdesivir being associated with arrhythmias and bradycardia [173].

On the other hand, others did not reproduce significant benefits and thus call into question the effectiveness of remdesivir. For example, one large trial that included 1,282 hospitalized patients found a decrease in in-hospital mortality, 60-day mortality, and the need for mechanical ventilation. However, these findings did not reach statistical significance [75]. Congruently, the DisCoVeRy trial did not reveal a significant difference in clinical status, time to hospital discharge, mortality, SARS-CoV-2 viral kinetics, or incidence of serious adverse events when using remdesivir [76].

Importantly, the WHO Solidarity trial was a large-scale RCT conducted multinationally and included a total of 14,221 adult COVID-19 patients whereby remdesivir showed no significant reduction in mortality for those patients who were already being ventilated. However, it had a small enhancing effect on reducing progression to ventilation, death, or both. This RCT's geographic scope makes it one of the most comprehensive trials done for COVID-19 treatments as it involved a total of 454 hospitals in 35 nations in all six of WHO's regions. In other words, although remdesivir was shown in the WHO Solidarity trial to modestly benefit patients on noninvasive oxygen support, it had not improved outcomes for severely ill and mechanically ventilated patients. As such, the study suggests that remdesivir should be considered on a selective basis for nonventilated, hospitalized patients while still standing as a moderately effective option with limited utility [77, 78]. An add-on RCT to the WHO Solidarity trial known as NOR-Solidarity, conducted in 2020, had evaluated the efficacy of remdesivir and hydroxychloroquine on in-hospital mortality from all causes in hospitalized COVID-19 patients. This RCT included a total of 185 patients from 23 hospitals in Norway but had concluded that remdesivir and hydroxychloroquine have no effect on viral clearance in hospitalized COVID-19 patients. The control group received the standard-of-care regimen. Nevertheless, these findings align with the WHO Solidarity trial's conclusions reflecting remdesivir's limited impact on recovery or mortality in COVID-19 patients, particularly those hospitalized [79].

The most recently published placebo-controlled, double-blind RCT conducted in 2023 on 1369 patients with mild-to-moderate COVID-19 had administered VV116, a novel agent manufactured from remdesivir with a dosage of 600 mg twice daily on Day 1 followed by 300 mg twice daily on Days 2–5. The trial revealed that VV116 reduced the median time to sustained resolution of clinical symptoms to 10.9 days as compared to 12.9 days observed for the placebo group (hazard ratio [HR] 1·21, 95% CI 1·04–1·40; *p* = 0·0023). These results reflect VV116's effectiveness and tolerability in COVID-19 patients with mild-to-moderate disease, supporting further trials to consolidate its use as a potential COVID-19 treatment [174].

### 4.2. Molnupiravir

The first double-blinded, placebo-controlled RCT conducted for molnupiravir in 2021 had reported a favorable safety profile along with promising pharmacokinetics and prompted further research for its effects on COVID-19 [80, 81]. This was followed by the MOVe-OUT trial in the same year with a larger patient population of 1433 participants, whereby molnupiravir significantly reduced the risk for hospitalization or death in at-risk COVID-19 adult patients with mild-to-moderate disease [84]. A secondary analysis of the trial's Phase 3 had shown a faster rate of normalization of the C-reactive protein and SpO2 as well as a reduced requirement for respiratory assistance and frequency of COVID-19–related acute care visits in the molnupiravir-treated patients compared to the placebo group, suggesting that molnupiravir's value extends beyond reduction in hospitalization or death [85]. In addition, 55 participants were considered immunocompromised, and data from the trial suggested increased benefits in treating mild-to-moderate COVID-19 adult patients who are immunocompromised and not hospitalized, emphasizing the observed effectiveness and safety [86]. In an investigation of the efficacy and exposure–response relationship of molnupiravir, the emphasis was drawn to the importance of patient characteristics in modulating treatment efficacy, especially considering baseline viral loads and molnupiravir's sustained viral control in decreasing adverse outcomes along with reducing the progression of commonly encountered COVID-19 symptoms [87, 88]. The MOVe-IN trial reported molnupiravir's effect in hospitalized COVID-19 patients, and unlike the MOVe-OUT trial, MOVe-IN had reported no observed clinical benefit. More importantly, these two trials highlighted the impact of avoiding a delay in time to treatment initiation in terms of symptom onset, especially before patients are hospitalized [91]. Of note, the MOVe-AHEAD trial highlighted molnupiravir's limited effectiveness as an agent for pre-exposure prophylaxis against COVID-19 in households without vaccinated members but are highly exposed [92]. Another study supported molnupiravir's safety profile and revealed its antiviral efficacy by presenting a reduction in viral RNA clearance time (14 days compared to the 15 days seen in the placebo group) and successful elimination of the virus by Day 5, further pushing for molnupiravir as a potential oral antiviral for early interventions in COVID-19 [89].

The AGILE CST-2 trial in the UK assessed the molnupiravir's efficacy in treating early COVID-19 in adult patients with mild-to-moderate disease split evenly between unvaccinated and vaccinated participants in addition to the fact that the patients were infected with a range of SARS-CoV-2 variants (delta, alpha, and omicron). This provided a broader scope for the efficacy of molnupiravir. The overall results revealed molnupiravir's ability to cause a greater reduction in viral load by Day 5, especially in vaccinated participants compared to placebo as well as a reduction in the viral clearance time, meaning that molnupiravir possesses moderate antiviral activity. However, the drug did not meet the required threshold for additional testing. This trial was the first to assess the effect of molnupiravir in vaccinated populations although its clinical benefit remained unclear at the time [93].

More importantly, the PANORAMIC trial is one of the largest studies done on molnupiravir. It tested the efficacy of molnupiravir in reducing occurrences of hospitalizations or death in adults with COVID-19 at high risk. The study included a total of 26,411 participants whereby the primary outcome of hospitalization or death had occurred almost equally in both the molnupiravir plus usual care and the usual care alone groups. Therefore, it was reported that although molnupiravir had effectively reduced the viral load and shortened time to recovery (median reduction of 4.2 days), it had no observed additional benefit in reducing hospitalizations or deaths in vaccinated, high-risk adults with COVID-19. As a result, the PANORAMIC trial implies that molnupiravir may assist in symptomatic recovery but does not recommend its use as the sole agent in a highly vaccinated population for reducing severe outcomes [94]. In a subsequent virology substudy under the PANORAMIC trial, conclusions about molnupiravir's effectiveness in initially decreasing the viral load showed that it can also lead to the prolongation of viral clearance time along with increased mutation rates, particularly after the cessation of the conventional 5-day treatment regimen. These results imply that the use of molnupiravir in highly vaccinated populations must be with caution due to risks of viral persistence and mutagenesis [95].

### 4.3. Favipiravir

Some RCTs report positive findings with favipiravir. In an open-label multiarm trial on COVID-19 positive patients, 1829 received favipiravir, 3256 received usual care, and 3726 received other treatments. Favipiravir was associated with a significantly shorter time to self-reported recovery compared to usual care (hazard ratio: 1.23, 95% CI: 1.14–1.33). However, hospitalization and mortality rates were similar across groups. [102]. Other studies showed significant association with shorter time to sustained clinical improvement [103] shortened time to the negativity of the nasopharyngeal swab [104]. On the other hand, two other trials each comprising 150 patients—one on hospitalized patients [105] and the other on patients with mild-to-moderate COVID-19 [106]—netted results that, while not significant, indicated increased viral clearance [105] and earlier RT-PCR negativity [106].

Other studies report no or limited benefit in using favipiravir. Viral clearance was not significantly faster [107–109], nor was time to symptom resolution [110]. Similarly, no significant differences in the requirement of oxygen therapy and risk to progression to severe disease [111], or in preventing hospitalization over a 28-day follow-up period when utilizing favipiravir [112]. In addition, disease progression from nonhypoxia to hypoxia was not prevented with early treatment [113] nor in shortening time to recovery [114]. The most frequent adverse effect associated with favipiravir is hyperuricemia [103, 105, 106]. Overall, while favipiravir may offer mild benefits in symptom reduction and viral clearance, its overall efficacy in treating COVID-19 is limited and inconsistent.

### 4.4. Baricitinib

Baricitinib was largely studied in patients hospitalized with COVID-19. One study analyzed its use in conjunction with dexamethasone and found that while statistical significance was not reached, there was a strong reduction in mortality by 28 days, which was maintained until 60 days [28]. Another study noted a reduction in the recovery time by one day and facilitation in improvement in clinical status, especially in patients receiving noninvasive ventilation [24]. A different study assessed baricitinib plus remdesivir compared to dexamethasone plus remdesivir in hospitalized patients for progression to mechanical ventilation or death, and found no significant difference in mechanical ventilation-free survival by Day 29 [29]. In critically ill patients receiving extracorporeal membrane oxygenation or invasive mechanical ventilation, mortality was found to be reduced with baricitinib, albeit this study was limited by a small sample size [26]. RECOVERY is the largest trial studying baricitinib, including 8156 patients hospitalized with COVID-19 in a randomized, controlled, open-label, platform trial. It was found to reduce 28-day mortality by 13%, increase the likelihood of discharge within 29 days, and decrease the risk of progression to invasive mechanical ventilation or death [27]. Other studies failed to show a significant reduction in disease severity with baricitinib [30] nor a survival benefit of baricitinib in patients with severe or critical COVID-19 [31]. One of the concerns surrounding baricitinib is whether it would result in secondary infections or thromboembolic events, but such adverse events were not significantly higher compared to controls [24, 26, 28].

### 4.5. Nirmatrelvir

A trial involving 2246 unvaccinated, nonhospitalized COVID-19 patients who are at high risk for severe disease, tested the use of nirmatrelvir boosted by ritonavir in a double-blind study. The risk of progression to severe COVID-19 was significantly reduced compared to placebo, with a relative risk reduction of 88.9% and 87.8% when commencing treatment within 3 days and 5 days of symptom onset, respectively [62]. A similar study on 1219 patients with mild-to-moderate COVID-19 in vaccinated and unvaccinated individuals did not find a significant difference in the time to alleviation of symptoms compared to placebo through the 28-day duration, hence the utility of the drug in patients not at high risk for severe COVID-19 having yet to be established [66]. Nirmatrelvir/ritonavir, when compared to molnupiravir in an open-label study on 209 low-risk patients with a high viral burden, reduced the viral load substantially more than molnupiravir, with molnupiravir's viral clearance being 25% slower [64]. Another study conducted during the omicron waves compared nirmatrelvir/ritonavir to intravenous sotrovimab and intramuscular tixagevimab/cilgavimab in 911 nonhospitalized patients with mild-to-moderate COVID-19 at risk for disease progression, of which 86% were vaccinated. Nirmatrelvir/ritonavir was found to be superior to the other two drugs in reducing hospital admission or death with an odds ratio (ORs) of 8.41 (*p* = 0.015) compared to the tixagevimab/cilgavimab arm and 2.42 (*p* = 0.499) compared to the sotrovimab arm [65]. Nirmatrelvir was also assessed as a potential postexposure prophylactic and whether it could reduce postacute sequelae of COVID-19; however, there was no significant difference in who developed symptomatic, confirmed infection compared to placebo [175]. A smaller study on 155 patients assessing nirmatrelvir/ritonavir's utility to reduce postacute sequelae such as body aches, fatigue, shortness of breath, and others found that at 10 weeks, there was no significant benefit compared to placebo [176].

### 4.6. Lopinavir

In an RCT published in 2020 and conducted on a total of 199 patients, the use of lopinavir/ritonavir in hospitalized adults with severe COVID-19 provided no benefit in the treatment group compared to standard care and relative to the time observed to clinical improvement. Additionally, gastrointestinal adverse events were more commonly identified in the lopinavir/ritonavir group [50]. Besides, the TOGETHER trial published in 2020 confirmed the absence of an observed benefit with either hydroxychloroquine or lopinavir/ritonavir in decreasing COVID-related hospitalization, time to the clearance of the virus, or time to hospitalization compared to placebo. All in all, both drugs did not perform a significant role in COVID-19 patients who were hospitalized and had advanced diseases [53]. A third RCT, Trial of Early Antiviral Therapies during Non-hospitalized Outpatient Window (TREAT NOW), published in 2021, had reported similar results to the prior two RCTs.

## 5. Antivirals and Pediatric Cardiac Patients

Although COVID-19 infection in children is generally less common and frequently presents with mild symptoms, there persists a risk of cardiac involvement, especially in those with congenital heart disease. Furthermore, in newborns and children who have undergone previous cardiac surgery, there is an increased risk of more severe disease, combined with an increased likelihood of requiring intensive care, intubation, and mechanical ventilation [177, 178]. A cohort study on 171,416 individuals between 2 months and 17 years old found that preexisting cardiovascular disease—including previous history of cardiac arrest or cardiogenic shock, heart surgery, cardiopulmonary disease, heart failure, nontraumatic cerebral hemorrhage, pericarditis, simple or complex biventricular defects, venous embolism and thrombosis, essential primary hypertension, and other hypertensive disorders—resulted in increased severity of COVID-19 to varying degrees. The highest ORs were associated with cardiac arrest (OR, 9.92; 95% CI, 6.93–14.20), cardiogenic shock (OR, 3.07; 95% CI, 1.90–4.96), and heart surgery (OR, 3.04; 95% CI, 2.26–4.08) [179].

As most trials analyzing COVID-19 treatment were studied in adults, treatment in the pediatric population is supportive and symptomatic [178]. In the context of pediatric patients, the use of antivirals in the treatment of COVID-19 has been routinely in practice in China. However, combination therapies like lopinavir/ritonavir are not currently recommended due to the lack of evidence from clinical trials regarding its efficacy and its discouraging pharmacodynamics [180, 181]. Moreover, remdesivir's preference over other antiviral medications stems from its observed beneficial effects and tolerance in adults with COVID-19. As such, although data about remdesivir's benefits in children with COVID-19 are lacking, the same assumption is made for pediatric patients. It is important to note that remdesivir was approved by the FDA in 2020 for use in adults and children that are 12 years of age and older, weighing 40 kg or more, and requiring hospitalization [181].

Cardiac side effects pertaining to various currently approved and investigational antiviral agents must be considered. When indicated, potential side effects of remdesivir, favipiravir, and lopinavir/ritonavir include QT prolongation with remdesivir also potentially causing bradycardia. Additionally, lopinavir/ritonavir can cause low-density lipoprotein (LDL) elevation leading to cardiovascular disease. Baricitinib's only contraindication was found to be its concomitant use with IL-6 inhibitors. However, molnupiravir and umifenovir were found to have no significant side effects [182].

## 6. Conclusion

The diversity in treatment guidelines across the globe increased with the urgency of the situation and the sheer volume of results coming out from observational studies and randomized controlled trials. With RCTs being the best source of evidence for evaluating the use of a drug, this process has led to certain drugs being approved in some countries and not in others, highlighting the importance of examining the study methodology. This includes evaluating the number of participants, their characteristics, location of the study, primary outcome and its measurements, and whether statistical significance truly corresponds to clinical significance. The current drugs that have been approved, namely, baricitinib, ensitrelvir, nirmatrelvir, remdesivir, and molnupiravir, have been studied with conclusions showing mixed results, while other novel antiviral medications have not been as extensively studied to make a conclusion on their efficacies. Although the pandemic was declared to have ended, the disease still circulates, calling for high-powered RCTs or the development of targeted therapy in addition to the repurposing of preexisting medications. Until then, the National Institutes of Health have issued treatment guidelines in order to direct decisions on the therapeutic management of COVID-19 in a variety of patients [183].

This population is at an increased risk for severe COVID-19 infection and has an increased mortality rate, with an increased risk of adverse outcomes with increased complexity of a congenital heart defect and worse NYHA functional class [184]. The pediatric population overall is also liable to unique complications following COVID-19 infection, particularly multisystem inflammatory syndrome in children (MIS-C) characterized by fever, hypotension or shock, cardiovascular dysfunction, coagulopathy, and acute gastrointestinal symptoms. Among the cardiovascular manifestations are myocardial dysfunction, pericarditis, valvulitis, and coronary abnormalities manifesting as echocardiography findings or elevated troponin or NT-proBNP [185, 186]. Further research is thus required on the understudied pediatric population, with studies evaluating the optimal antiviral in the treatment of acute COVID-19 in the children and the potential to reduce the risk of long-term postviral cardiovascular complications. Such research should prioritize the vulnerable population of children with preexisting cardiac conditions, considering safety profiles, potential for drug–drug interactions, efficacy, and mitigation of postviral cardiac dysfunction.

## Figures and Tables

**Table 1:** An overview of antivirals used in COVID-19.

Drug class	Drug	Mechanism of action	Current indication	Date of earliest use in COVID-19	Demonstrated efficacy in COVID-19
Fusion inhibitors	Baricitinib [23–25]	JAK1/2 inhibitor JAK is a tyrosine kinase involved in the phosphorylation of downstream intracellular proteins that subsequently bind to the STAT transcription factor, which then translocates to the nucleus to mediate the expression of genes involved in immunity and inflammation. Baricitinib thus provides an anticytokine effect. Moreover, it is important to note that the endocytosis of SARS-CoV-2 following the binding of the S protein to ACE2 is mediated by the NF-κB activating kinase (NAK) family of signaling molecules, after which the virus is able to proliferate. By inhibiting NAK activity, baricitinib ensures the inhibition of viral proliferation and successive viral transmission between cells [23]	Treatment of moderate-to-severe rheumatoid arthritis unresponsive to other DMARDs Treatment of COVID-19 in hospitalized patients and standalone treatment for adult patients Treatment of alopecia areata Off-label: Treatment of atopic dermatitis, psoriatic arthritis, and vitiligo	December 2020	Yes: [26, 27] No: [24, 28–31]
	Umifenovir [32–38]	Nonnucleoside fusion inhibitor It prevents either the fusion of the virus with the endosome or its binding to host cells by affecting the hydrogen bonds of host cell membrane's phospholipid molecules, whereby umifenovir performs dual binding to ACE2 and the receptor-binding domain of the SARS-CoV-2 S protein [32, 33]. Moreover, umifenovir was shown to modify the acidification process within the endocytic vesicle, inhibiting viral particle release intracellularly [33]	Licensed in China and Russia for the treatment of respiratory viral infections including influenza virus Under investigation for treatment and prophylaxis of COVID-19	February 2022	Yes: [37^1^, 39] No: [37^1^, 40]
	Camostat mesylate [41–45]	TMPRSS2 inhibitor TMPRSS2 is a Type II transmembrane serine protease, which is widely expressed inside epithelial cells. This protease facilitates the cellular entry of several viruses, among which are SARS, MERS, and influenza. TMPRSS2 cleaves the spike protein at the 2′ site, which is understood as a step that activates the spike protein's membrane fusion activity. Thus, TMPRSS2 is a critical mediator of viral entry. Camostat mesylate binds to the protease's active site where it is cleaved into its active form known as GBPA and forms a covalent bond, not only competitively inhibiting TMPRSS2 but also providing substrates leading to its covalent inhibition [41]	Treatment of chronic pancreatitis in Japan Treatment of drug-induced lung injury in Japan Under investigation as potential treatment for COVID-19	January 2023	No: [44, 46–49]

Protease inhibitors	Lopinavir [50–52]	Antiretroviral protease inhibitor	Used in combination with ritonavir (collectively called Kaletra) for the treatment of HIV-1 in adults and pediatric patients that are 14 days and older Not FDA-approved for COVID-19 treatment yet	May 2020	No: [50, 53, 54]
	Ensitrelvir (Xocova) [55–57]	Direct M-pro inhibitor	Treatment of COVID-19 in Japan Under investigation for the treatment of COVID-19 outside Japan	February 2023	Yes: [58, 59] No: [60]
	Nirmatrelvir/Ritonavir [61–63]	Direct M-pro inhibitor	Treatment of mild-to-moderate COVID-19 in adults at high risk of progression to severe COVID-19 (i.e., hospitalization or death)	April 2022	Yes: [62, 64, 65] No: [66]

Nucleoside or nucleotide analogs	Remdesivir [67–69]	Adenosine NA; delayed chain termination Delayed chain termination encompasses remdesivir's ability to incorporate first and moves upstream to a site prior to the primer strand where it then carries out termination [70]. Remdesivir is an adenosine analog with a 1′-ribose cyanosubstitution whereby its active triphosphate form (RDV-TP) stalls the RdRp for three nucleotides following its incorporation, which means it allows the addition of three more nucleotides following which nucleotides cannot be added. The preferential RDV-TP incorporation over the natural ATP together with remdesivir's ability to slow the polymerization rate are thought to be contributing to remdesivir's antiviral effect. The most important activity of remdesivir though remains its ability to act as a chemical corruptor owing to its 1′-cyano group modification which influences RNA folding [67]	Treatment of COVID-19 in hospitalized adult and pediatric patients (≥ 28 days and ≥ 3 kg) Treatment of nonhospitalized patients with mild-to-moderate COVID-19 at high risk of progression to severe COVID-19 (i.e., hospitalization or death) 2020 EUA: FDA-approved for use in children and adults with suspected or confirmed COVID-19 in hospital settings with SpO2 ≤ 94% Revised EUA: Approved for use in hospitalized pediatric patients weighing between 3.5 and 40 kg and pediatric patients < 12 years old and weigh ≤ 3.5 kg with laboratory-confirmed or suspected COVID-19 Patients without need for ECMO or invasive mechanical ventilation must be treated for 5 days, which may be extended to 10 days if no improvement Patients requiring ECMO or invasive mechanical ventilation must be treated for 10 days Europe: Approved for the treatment of COVID-19 in adults and adolescents (≥ 40 kg) with pneumonia that requires oxygen supplementation; also indicated for COVID-19 treatment in adults without the requirement of supplemental oxygen but are at high risk of progression to severe COVID-19	May 2020	Yes: [71–74] No: [68, 75–79]
	Molnupiravir [80–83]	Isopropyl ester cytidine NA; performs error catastrophe in RdRp Molnupiravir is a prodrug that is cleaved by host plasma esterases into the active NA, after which it diffuses into several tissues and is converted to its triphosphate form. Consequently, it inhibits SARS-Cov-2's RdRp and reduces the viral replication. Molnupiravir is also thought to be effective in treating patients who developed resistance to remdesivir [82]	United States: Emergency treatment of mild-to-moderate COVID-19 adults at high risk Europe: Treatment of mild-to-moderate COVID-19 in high-risk patients (i.e., prevent progression to hospitalization or death)	April 2021	Yes: [80, 81, 84–90] No: [91–95]
	Favipiravir [96–101]	Purine base NA prodrug Favipiravir is a purine base NA that is converted by host cell phosphoribosylation into the active form: favipiravir ribofuranosyl-5B-triphosphate. The active drug acts as a potent and selective inhibitor of RdRp by becoming incorporated into the growing viral RNA, leading to chain termination and viral mutagenesis. The significance of favipiravir as a mutagen is that this feature allows it to escape the repair machinery of coronavirus [96].	Japan: Approved for treatment of patients with influenza that are unresponsive to standard treatment Under investigation for the treatment of Ebola virus infection and COVID-19	October 2020	Yes: [102–106] No: [107–115]
	Ribavirin [116–119]	Guanosine NA; mutagen; mRNA capping Ribavirin is a guanosine NA originally used for treating Hepatitis C virus (HCV) infections and has broad-spectrum activity as an antiviral against both DNA and RNA viruses. Potential mechanisms by which it acts include the inhibition of the viral RNA polymerase, mRNA capping, and as a mutagen during viral replication [116]	Treatment of HCV infection typically in combination with other antiviral drugs with the goal to cure or achieve an SVR Added to reduce relapse rates and reduce SVR In TECHNIVIE therapy indicated in the treatment of HCV Genotypes 1a and 4 infections: Addition of ribavirin is recommended in patients with or without cirrhosis	May 2020	Yes: [120]
	Sofosbuvir/daclatasvir/ledipasvir/velpatasvir (Epclusa) [67, 70, 121–124]	Uridine NA; immediate chain termination Immediate chain termination as seen with the uridine analog sofosbuvir is characterized by effective competition occurring between the NA and RdRp's natural NTP, thereby achieving successful termination following NA incorporation into the primer strand [70]. Due to the absence of a 3′-hydroxyl group from the incorporated NA, the addition of any more downstream nucleotides is inhibited at the active site of the viral polymerase [67]. More recent studies revealed sofosbuvir's active form 2′-F-Me-UTP has the ability to maintain its activity at the catalytic site, termed the +1 site of the SARS-CoV-2 RdRp [70].	Used in combination with other antiviral drugs for the treatment of chronic HCV infections (HCV Genotypes 1–6) and in the treatment of HCV and HIV coinfected patients Daclatasvir: This combination is indicated with or without ribavirin for the treatment of chronic HCV infections of Genotypes 1a/b or 3/ledipasvir: This combination is indicated in the treatment of HCV with any of Genotypes 1 and 4–6 infections with compensated cirrhosis or without cirrhosis/ledipasvir with ribavirin: Indicated for the treatment of decompensated cirrhosis in Genotype 1 infection or the treatment of Genotype 1 or 4 infected liver-transplant patients with compensated cirrhosis or without cirrhosis/velpatasvir: This combination (Epclusa) is indicated for the treatment of adult patients with chronic HCV any of Genotypes 1–6 infection with compensated cirrhosis or without cirrhosis; indicated with ribavirin in patients with decompensated cirrhosis	November 2020	Sofosbuvir/ledipasvir Yes: [125, 126] Sofosbuvir/velpatasvir No: [127] Sofosbuvir/ravidasvir No: [128] Sofosbuvir/daclatasvir Yes: [128] No: [129–131]

M2 ion channel blockers	Amantadine [17, 132–134]	Protein-membrane channel inhibitor	Chemoprophylaxis, prophylaxis, and treatment of infection caused by several strains of Influenza A virus Treatment of parkinsonism Treatment of drug-induced extrapyramidal reactions	June 2023	Yes: [134, 135] No: [136]

Abbreviations: COVID-19, coronavirus disease 19; DMARDs, disease-modifying antirheumatic drugs; ECMO, extracorporeal membrane oxygenation; EUA, Emergency Use Authorization; FDA, Food and Drug Administration; HCV, Hepatitis C virus; HIV-1, human immunodeficiency virus 1; JAK, Janus kinase; M-pro, main protease; NA, nucleoside or nucleotide analog; RdRp, RNA-dependent RNA polymerase; SVR, sustained virologic response; TMPRSS2, transmembrane serine protease.

^1^based on patient characteristics.

**Table 2:** Pharmacology and adverse effects associated with selected antivirals.

Drug class	Drug	ROA	Dose and duration	Bioavailability	Metabolism	Half-life	Elimination	Adverse effects
Fusion inhibitors	Baricitinib [137]	Oral	2 or 4 mg o.d. 7–14 days	79%	Liver: 6% by CYP3A4	6–9 h	Renal	Upper respiratory tract infection, headache, nasopharyngitis, lymphopenia, thrombosis, opportunistic infection, malignancy, gastrointestinal perforations, cardiovascular events
	Camostat mesylate [138–141]	Oral, IV	100–300 mg t.i.d. p.o.	5%	Carboxylesterases: Camostat is converted rapidly to GBPA. Not metabolized by the CYP-p450 enzymes or liver metabolism	GBPA: < 2 h	Renal	Rash and pruritus, nausea, abdominal discomfort, liver enzyme elevation
	Umifenovir [37, 142]	Oral	800 mg b.i.d.		CYP3A4, FMO family, and UDP family in hepatic and intestinal microsomes	16 h		Headache, nausea, stomachache, vomiting

Protease inhibitors	Ensitrelvir [143, 144]	Oral	125–250 mg o.d.		Inhibits CYP3A	42.2–48.1 h	Renal	Decreased HDL, diarrhea, headache, eczema
	Lopinavir/Ritonavir [145–147]	Oral	400/100 mg b.i.d.	25% in the absence of ritonavir	Liver: Lopinavir is metabolized by CYP3A4 and CYP3A5. Ritonavir inhibits CYP3A4, CYP2D6, or P-gp	2–3 h after a single dose and 4–6 h after multiple doses	Fecal Very limited renal excretion	Diarrhea, nausea, vomiting, immune reconstitution syndrome, pancreatitis, hypertriglyceridemia, hypercholesterolemia, fat redistribution
	Nirmatrelvir/Ritonavir [146, 148]	Oral	300/100 mg b.i.d. for 5 days, with 150/100 mg for those with moderate renal impairment		Ritonavir inhibits CYP3A4, CYP2D6, or P-gp.	6.8–8 h	Renal	Dysgeusia, bloating, diarrhea, nausea, vomiting, allergy, headache

Nucleoside or nucleotide analogs	Favipiravir [149–152]	Oral	1600–1800 mg b.i.d on Day 1, 600–800 mg b.i.d. from Day 2 2–14 days	94%	Liver: aldehyde oxidase, xanthine oxidase Not metabolized by the CYP-p450 systems but inhibits CYP2C8	2.5 h	Renal	Hyperuricemia, liver dysfunction, diarrhea, nausea, abdominal pain, thrombocytopenia
	Molnupiravir [153–156]	Oral	400 or 800 mg b.i.d		Cellular metabolism to uridine and/or cytidine via endogenous pyrimidine metabolism	7 h	The renal system is not a meaningful site of elimination	Headache, diarrhea, malaise, nausea
	Remdesivir [149, 157, 158]	IV	200 mg o.d. Maintenance dose of 100 mg for up to 10 days	100%	Liver: Carboxylesterase 1, Cathepsin A, CYP3A	Prodrug: 1 h Active metabolite: 43 h	Renal	Infusion site phlebitis, respiratory toxicity, headache, hepatotoxicity, nausea, diarrhea, vomiting, rash, multiple organ dysfunction syndrome, deep vein thrombosis, delirium, septic shock, pyrexia
	Ribavirin [159–161]	Oral, IV	2 or 4 g p.o. Loading dose, with a maintenance dose of 0.6–1.2 g p.o. q8h for 4–10 days Alternatively, 500 mg IV b.i.d. or t.i.d.	52%		37 h	Renal	Hemolytic anemia, itching, rash, cough, neuropsychiatric side effects like insomnia
	Sofosbuvir [162]	Oral	400 mg o.d.	≥ 80%	Hydrolase cleavage, with CYP-p450, FMO enzymes, and UGTs	18 h	Renal	Fatigue, headache, nausea, insomnia, or irritability

M2 ion-channel blockers	Amantadine [163–165]	Oral	100 mg b.i.d.	Believed to be nearly complete		16 h	Renal	Weight loss, anorexia, insomnia, constipation, dizziness, blurred vision, urinary straining, livedo reticularis, dryness of mouth, abdominal discomfort, edema, tiredness

Abbreviations: CYP-p450, cytochrome p450; FMO, flavin-containing mono-oxygenase; GBPA:, 4-(4-guanidinobenzoyloxy) phenylacetic acid; HDL, high-density lipoprotein; P-gp, p-glycoprotein; ROA, route of administration; UGT, glucuronosyltransferase.

**Table 3:** High-impact studies that demonstrated efficacy of antivirals in COVID-19.

Study	Study type	Site and date	Study size	Antiviral agent and dosage	Primary outcome
Molnupiravir for oral treatment of COVID-19 in nonhospitalized patients [84–88, 90] (MOVe-OUT trial)	Phase 3, randomized, double-blind, placebo-controlled trial	> 20 sites, 2021 including the United States, Canada, Brazil, Argentina, the United Kingdom, France, Germany, Italy, Poland Spain, Japan, Philippines, Malaysia, and South Africa	1433 nonhospitalized, unvaccinated adults exhibiting mild-to-moderate symptoms or confirmed COVID-19 for ≤ 5 days	Molnupiravir: 800 mg twice daily for 5 days	Hospitalization or death on Day 29: Recorded in 6.8% of the molnupiravir group versus 9.7% of the placebo group
Remdesivir for the treatment of COVID-19—final report [71]	Phase 3, randomized, double-blind, placebo-controlled trial	60 sites, 2020 including the United States, Denmark, the United Kingdom, Greece, Germany, Korea, Mexico, Spain, Japan, and Singapore	1062 hospitalized confirmed COVID-19 patients with evidence of lower respiratory tract involvement	Remdesivir: Intravenous 200 mg loading dose on day 1 and then 100 mg once daily for up to 10 days	Recorded time to recovery was 10 days in the remdesivir group compared to the placebo group's 15 days
Baricitinib in patients admitted to hospital with COVID-19 (RECOVERY): a randomized, controlled, open-label, platform trial and updated meta-analysis [27]	Randomized, controlled, open-label, platform trial	UK, 2021	8156 patients hospitalized with COVID-19	Baricitinib 4 mg daily for 10 days	Reduction in 28-day all-cause mortality by one-eighth
Oral nirmatrelvir for high-risk, nonhospitalized adults with COVID-19 [62]	Randomized controlled, double-blind, Phase 2–3, placebo-controlled	United States, 2021	2246 patients that were unvaccinated, nonhospitalized, and at high risk for progression to severe COVID-19	Nirmatrelvir/ritonavir 300/100 mg every 12 h for 5 days	88.9% and 87.8% relative risk reduction of COVID-19–related hospitalization or death from any cause through Day 28 when starting treatment within 3 days and within 5 days after symptom onset, respectively
Efficacy and safety of 5-day oral ensitrelvir for patients with mild to moderate COVID-19: The SCORPIO-SR randomized clinical trial [59]	Randomized controlled, double-blind, Phase 3 part of Phase 2–3, placebo-controlled	Japan, Vietnam, and South Korea, 2022	1821 patients with mild-to-moderate COVID-19, with 91.0% of patients vaccinated with two or more doses	375 mg ensitrelvir on Day 1 and 125 mg on Days 2–5 or 750 mg ensitrelvir on Day 1 and 250 mg on Days 2–5	Treatment with ensitrelvir reduced symptom duration by about one day compared to placebo, decreasing the median duration from 5 days to 4 days

**Table 4:** High-impact studies that demonstrated no effect or a negative effect of antivirals in COVID-19.

Study	Study type	Site and date	Study size	Antiviral agent and dosage	Primary outcome
Remdesivir and three other drugs for hospitalized patients with COVID-19: Final results of the WHO Solidarity randomized trial and updated meta-analyses [77, 78]	Randomized, controlled, multinational, multicenter, adaptive trial	454 hospitals in 35 countries, 2021	14,221 adult participants ≥ 18 years old with confirmed COVID-19 irrespective of disease severity	Remdesivir: Intravenous 200 mg loading dose on Day 0 and then 100 mg daily from Days 1 to 10 Hydroxychloroquine: orally four tablets at Hour 0 and then four tablets at Hour 6 followed by two tablets at Hour 12 and every 12 h for 10 days (each tablet contained 200 mg hydroxychloroquine sulfate) Lopinavir/ritonavir: Orally 2 tablets twice daily for 14 days where each tablet contained 200 mg lopinavir and 50 mg ritonavir Interferon Beta-1a: 44 μg subcutaneously on Days 0, 3, and 6	In-hospital mortality (irrespective of before or after 28 days) recorded as 14.5% for remdesivir compared to 15.6% for control
Remdesivir plus standard of care versus standard of care alone for the treatment of patients admitted to the hospital with COVID-19 (DisCoVeRy): a Phase 3, randomized, controlled, open-label trial [76]	Randomized, controlled, open-label, Phase 3	France, Belgium, Austria, Portugal, and Luxembourg, 2021	857 patients that were hospitalized with SARS-CoV-2 infection, presenting with at least one of the following: rales or crackles on examination, oxygen saturation ≤ 94%, or requirement of supplemental oxygen or ventilation	IV remdesivir 200 mg on Day 1, then 100 mg 1-h infusion once daily for a total duration of 10 days	The distribution of the seven-point ordinal scale^1^ on Days 15 and 29 showed no significant difference between the remdesivir and control groups
Molnupiravir plus usual care versus usual care alone as early treatment for adults with COVID-19 at increased risk of adverse outcomes (PANORAMIC): An open-label, platform-adaptive randomized controlled trial [94]	Randomized, controlled, open-label, national, multicenter, prospective, platform-adaptive trial	UK, 2022	26,411 participants ≥ 50 years old or ≥ 18 years old with relevant comorbidities who had been feeling unwell with confirmed COVID-19 for ≤ 5 days	Molnupiravir: 800 mg twice daily for 5 days plus usual care	All-cause, nonelective hospitalization or death at 28 days postrandomization: Recorded in 1% (105) of the molnupiravir plus usual care group versus 1% (98) of the usual care alone group
Favipiravir in patients hospitalized with COVID-19 (PIONEER trial): a multicenter, open-label, Phase 3, randomized controlled trial of early intervention versus standard care [114]	Randomized, controlled, open-label, Phase 3	UK, Brazil, and Mexico, 2021	499 patients that were hospitalized for suspected or confirmed COVID-19	Favipiravir 1800 mg twice daily on Day 1, then 800 mg twice daily from Day 2 to Day 10	There was no significant difference in time to recovery according to a seven-category ordinal scale^2^ from randomization to Day 28
Nirmatrelvir for vaccinated or unvaccinated adult outpatients with COVID-19 [66]	Randomized controlled, double-blind, Phase 2–3, placebo-controlled	United States, 2022	1296 patients that were either unvaccinated or had not been vaccinated within the past year or fully vaccinated with a high risk of progression to severe COVID-19	Nirmatrelvir/ritonavir 300/100 mg every 12 h for 5 days	There was no significant difference in efficacy compared to placebo, assessed by the time difference to sustained relief of all specified COVID-19 signs and symptoms up to Day 28
Evaluation of the effect of sofosbuvir and daclatasvir in hospitalized COVID-19 patients: a randomized double-blind clinical trial (DISCOVER) [130]	Randomized, controlled, double-blind, placebo-controlled	Iran, 2020	1083 patients hospitalized with COVID-19	Sofosbuvir/daclatasvir 400/60 mg once daily for 10 days	Sofosbuvir/daclatasvir showed no significant impact on hospital discharge rates or survival compared to placebo
Effect of early treatment with hydroxychloroquine or lopinavir and ritonavir on risk of hospitalization among patients with COVID-19: The TOGETHER randomized clinical trial [53]	Randomized, controlled trial	Brazil, 2020	685 patients with less than 8 days reported onset of flulike symptoms or chest computerized tomography scan indicating COVID-19	Hydroxychloroquine: Loading dose of 800 mg, then 400 mg/day for 9 days Lopinavir/ritonavir: Loading dose of 800 mg and 200 mg, respectively, every 12 h, then 400 mg and 100 mg, respectively, every 12 h for 9 days	COVID-19–related hospitalization and death at 90 days after randomization Hospitalization recorded in 3.7% (8) of the hydroxychloroquine group, 5.7% (14) of the lopinavir/ritonavir group, and 4.8% (11) of the placebo group Death recorded in one participant of the placebo group and two participants in the lopinavir/ritonavir group

^1^Seven-point ordinal scale (Version 3.0, March 3, 2020 WHO Master Protocol): 1, not hospitalized, no limitation on activities. 2, not hospitalized, limitation on activities. 3, hospitalized, not requiring supplemental oxygen. 4, hospitalized, requiring supplemental oxygen. 5, hospitalized, on noninvasive ventilation or high flow oxygen devices. 6, hospitalized, on invasive mechanical ventilation or ECMO. 7, dead.

^2^Seven-category ordinal scale: 1, not hospitalized and able to resume normal activities; 2, not hospitalized but unable to return to normal activities; 3, hospitalized without the need for supplemental oxygen; 4, hospitalized and requiring supplemental oxygen; 5. hospitalized and needing nasal high-flow oxygen therapy, noninvasive ventilation, or both; 6, hospitalized and requiring extracorporeal membrane oxygenation, invasive mechanical ventilation, or both; 7, death.

**Table 5:** Summarized RCT findings on various antivirals.

Drug class	Drug	Findings
Fusion inhibitors	Camostat mesylate	When tested in hospitalized patients [46, 47] or in patients with mild-to-moderate COVID-19 [48, 49], camostat mesylate was not found to improve clinical outcomes
		One study conducted in patients with mild-to-moderate COVID-19 found a clinical improvement in patients taking camostat mesylate, albeit it was not statistically significant [44]
	Umifenovir	When studied in asymptomatic patients or those with mild COVID-19 in a double-blind trial with a total of 132 patients, umifenovir significantly reduced time to RT-PCR nasopharyngeal swab negativity. In addition, patients with moderate COVID-19 improved faster than patients on placebo, but this was not statistically significant [37]
		An open-label trial comparing lopinavir/ritonavir to umifenovir in 100 hospitalized patients in combination with hydroxychloroquine found that umifenovir improved a variety of clinical and laboratory outcomes, including peripheral oxygen saturation levels, need for ICU admission, length of hospital stay, chest CT findings, white blood cell count (WBC), and erythrocyte sedimentation rate (ESR) [39]
		A study evaluating umifenovir monotherapy compared to lopinavir/ritonavir monotherapy in 86 patients hospitalized with mild-to-moderate COVID-19 did not find any benefit in either [40]

Protease inhibitors	Ensitrelvir	In 341 patients with mild-to-moderate COVID-19, one study found a decrease in viral titer on Day 4 albeit no significant change in symptoms [58]
		A study including 1821 patients found that, when initiated in less than 72 h of disease onset, ensitrelvir shortened the time needed for the resolution of the five common COVID-19 symptoms (nasal congestion, sore throat, cough, fatigue, and feverishness) compared to a placebo, albeit by around one day [59]
		In asymptomatic or mild COVID-19, a nonsignificant improvement was noted, along with a reduction in viral load, in a trial including 572 participants [60]
		991 patients who were treated with two different doses of ensitrelvir or placebo were self-assessed for the development of post-COVID-19 condition (PCC), characterized by a wide array of symptoms ranging from fatigue to cognitive dysfunction to the persistence of acute-phase symptoms such as fever, with results showing a risk reduction compared to placebo [171]

Nucleoside or nucleotide analogs	Ribavirin	Ribavirin was studied in isolation in an open-label trial comparing sofosbuvir/daclatasvir to ribavirin in 62 patients with signs of severe COVID-19, where the researchers found the former to be superior to the latter in terms of shortening duration in the hospital, duration in the CI, and mortality rate, along with fewer side effects such as anemia and GI bleeding [120]
		One study compares Interferon Beta-1b, lopinavir/ritonavir, and ribavirin versus lopinavir/ritonavir alone in an open-label trial on 127 hospitalized patients. Triple therapy was found to be superior in relieving symptoms, reducing viral shedding duration, and shortening hospital stays in patients with mild-to-moderate COVID-19, with minor gastrointestinal side effects including diarrhea and vomiting. Due to the nature of the study, it is unclear whether the benefit was due to synergism or due to either ribavirin or Interferon Beta-1b alone [119]
		A study analyzed whether sofosbuvir/daclatasvir combined with ribavirin in hospitalized patients with moderate COVID-19 produces any benefit relative to standard of care, but there was no significant difference in length of hospital stay, ICU admissions, or number of deaths. However, the cumulative incidence of recovery was significantly higher in the treatment group. With a sample of 48 patients with imbalanced baseline characteristics, the study is too small to make a definitive conclusion [123]
	Sofosbuvir	An open-label trial involving 82 patients with mild-to-moderate COVID-19 evaluated sofosbuvir/ledipasvir and found that the drug combination achieved the clinical response more quickly. Furthermore, no differences were observed in hospital and ICU stay or 14-day mortality [125]
		The same combination was studied compared to oseltamivir, hydroxychloroquine, and azithromycin in 250 patients with moderate COVID-19 in a single-blinded study, where the sofosbuvir/ledipasvir had a significantly higher cure rate by twofold [126]
		The combination of sofosbuvir/velpatasvir, when utilized in 80 patients with moderate-to-severe COVID-19 in an open-label study, did not significantly reduce the 28-day mortality rate, nor was there any difference in mortality, time to clinical improvement, RT-PCR conversion, and necessity for and time free from mechanical ventilation compared to controls [127]
		In an open-label, not placebo-controlled trial, sofosbuvir plus ravidasvir or daclatasvir compared to standard of care were studied in 120 patients with moderate or severe COVID-19. While outcomes did not vary significantly with the ravidasvir combination, sofosbuvir/daclatasvir netted a significantly lower amount of symptoms including fever, respiratory distress, headache, and generalized aches with no signs of worsening such as ICU admission or mechanical ventilation on the seventh and tenth days [128]
		A smaller open-label study on 66 patients with moderate or severe COVID-19 did not find a survival benefit—assessed as clinical recovery within 14 days of initiating treatment—using the same dosages of sofosbuvir/daclatasvir of 400/60 mg [129]
		The DISCOVER trial, involving 1083 patients hospitalized with COVID-19 in a placebo-controlled double-blind clinical trial, similarly assessed sofosbuvir/daclatasvir and observed no differences in time to hospital discharge nor time to in-hospital mortality [130]
		A trial considering the use of sofosbuvir/daclatasvir in 55 patients with mild COVID-19 found no significant alleviation of symptoms 7 days after initiating treatment, albeit this study did not utilize an objective grading system and could not utilize viral load tests to measure treatment responses due to governmental restrictions. Of note, the drug combination did lead to a significant reduction in the percentage of patients with fatigue and dyspnea after 1 month [131]
		Sofosbuvir/daclatasvir was utilized in an open-label trial of 828 patients to study its ability to prevent SARS-CoV-2 infection among healthcare workers or other high-risk individuals and found there was no significant preventative effect [172]

M2 ion-channel blockers	Amantadine	Amantadine, when studied in nonhospitalized patients over 40 years or adult patients with at least one comorbidity, did not affect disease progression [135]
		A study utilizing a population of 99 unvaccinated patients with mild-to-moderate COVID-19 found a significant improvement in clinical status, with a noteworthy improvement in mood and arousal to correlate with amantadine's potential CNS effects potentially ameliorating COVID-19's neuropsychiatric manifestations [136]
		In a study on hospitalized adults, amantadine did not show clinical efficacy, albeit an interim analysis on 150 patients showing no trend toward improvement resulted in early termination of the trial [134]

## Data Availability

Data sharing is not applicable to this article as no new data were created or analyzed in this study.
